# No clear evidence of PFAS-induced immunomodulation in SARS-CoV-2-specific human T cell responses following vaccination

**DOI:** 10.1007/s00204-026-04511-y

**Published:** 2026-07-23

**Authors:** Hermann-Josef Thierse, Katharina Sommerkorn, Moustapha Sy, Patrick Tarnow, Andreas Luch, Katherina Siewert

**Affiliations:** 1 Department of Chemicals and Product Safety, German Federal Institute for Risk Assessment (BfR), Berlin, Germany; 2 Department Food and Feed Safety in the Food Chain, German Federal Institute for Risk Assessment (BfR), Berlin, Germany

**Keywords:** Per- and polyfluoroalkyl substances (PFAS) exposure, Severe acute respiratory syndrome corona virus 2 (SARS-CoV-2), COVID-19 vaccination, Human peripheral blood mononuclear cells (PBMC), Antigen-specific T cell activation, CD154

## Introductory remarks

The human organism is exposed to an armada of distinct per- and polyfluoroalkyl substances (PFAS). PFAS and related chemical substances are found in drinking water and many consumer products (FDA 2026; National Academies of Sciences 2022), and they have an increasingly critical impact on the environment and human health (BfR 2023; EFSA et al. 2020; Health-Canada 2025). For example, it has been observed that, under certain vaccination conditions—such as the type of vaccine (e.g. tetanus, diphtheria) and the vaccination schedule under investigation—PFAS in maternal serum may have triggered a significant immunosuppressive humoral effect in young children (Grandjean et al. 2012). Although the humoral immunomodulatory effects of PFAS are still under debate (Antoniou et al. 2022; Bline et al. 2024; Ehrlich et al. 2023; Fenton et al. 2021), it appears that the in vitro activation of human T cells can also be influenced by PFAS (Maddalon et al. 2023).

In a recent in vitro study published in Environment International, Ayuk et al. (2025) investigated whether PFAS exposure to human peripheral blood mononuclear cells (PBMCs) from SARS-CoV-2 (Severe Acute Respiratory Syndrome-Coronavirus-2) immunized and infected individuals affected the in vitro response to a SARS-CoV-2_S (spike) protein peptide pool. Blood donors included 5 women and 5 men, all vaccinated with mRNA vaccines from Pfizer-BioNTech (BNT162b2 or Comirnaty™) or Moderna (mRNA-1273) encoding the SARS-CoV-2 spike protein of the Wuhan strain. Some of the donors also had documented infection(s) with SARS-CoV-2. With regard to possible PFAS-related adverse effects on immune functionality in association with vaccine responses, the authors concluded that “a significant upregulation of interferon-gamma (IFN-γ) production in T and NK cells was observed, particularly in male donors exposed to high concentrations of the PFAS mixture”, and “a decrease in the total B-cell population, particularly among female donors”. They observed that exposure to the selected mixture of PFAS reduced macrophage inflammatory protein-1 alpha (MIP-1α/CCL3) and macrophage inflammatory protein-3 alpha (MIP-3α/CCL20) levels, which could indirectly lead to “insufficient T-cell and B-cell crosstalk for optimal antibody production”. Although the study could provide new insights into some potential immunomodulatory effects of PFAS on human immune responses to SARS-CoV-2-spike protein, doubts about the conclusions are raised by some fundamental questions concerning the study design, the methodology, and the interpretation of the data, including the immunomodulatory conclusions drawn.

## Basic requirements and limitations for the activation of SARS-CoV-2-specific human T cells

### Key limitations of the experimental design for re-stimulation of SARS-CoV-2-reactive human T cells

Similar to the authors (Ayuk et al. 2025), Dörnte et al. (2022) also used SARS-CoV-2-derived peptides from the same ‘mega pool of 360 15-mer peptides covering the complete sequence of the wild-type Spike protein’ (‘PepTivator® SARS-CoV-2 Prot_S Complete, Miltenyi Biotec, Cat. No. 130-127-951). While Dörnte et al. (2022) used the peptide pool to compare the T cell responses of the original Wuhan strain and the mutated B.1.1.529 (Omicron) strain of SARS-CoV-2, differences in the activation protocol, controls used, and reporting of data relative to Ayuk et al. (2025) all raise questions about the strength of conclusions by Ayuk et al. (2025).

#### Sample size and characterization of vaccinated blood donors

When drawing conclusions about SARS-CoV-2 immunity (and the associated effects of PFAS) in humans, the sample size (5 women and 5 men) of the study by Ayuk et al. (2025) poses a significant problem, particularly with regard to the question of sex-specific effects—a point that is even raised by the authors themselves. We agree with the authors that the sample size in this study is very small. Thus, to us it is obvious that with n = 5, no robust statistical conclusions can be drawn regarding the sex-specific variable NK-, T- and B-cell-specific immune responses to SARS-CoV-2 vaccination or a potential impact on vaccine efficacy. Even within each sex, there is often significant variation among the 5 donors, such as three male responders that show increased IFN-γ production in NK cells while the other two male donors do not respond (100× and 1000× PFAS concentrations). However, even if there would be a sex-specific trend within the low numbers, this could be expressed more cautiously (Corsini et al. 2012). The studies by Ferroni et al. (2024) and Trevisan et al. (2023) provide data on SARS-CoV-2 vaccinations and sex-specific differences; however, Ferroni et al. included 25,893,992 vaccinated and 15,782,591 unvaccinated individuals, as well as 1,175,804 SARS-CoV-2 infections and 51,531 severe COVID-19 cases, whilst Trevisan et al. examined sex-specific differences in the efficacy and safety of SARS-CoV-2 vaccination among residents of long-term care facilities (LTCFs; N = 3259). In other words, a larger number of donors is usually required to reliably detect sex-specific SARS-CoV-2 immune responses. With respect to the authors’ analyses (Ayuk et al. 2025) (correlations in Supplementary Materials), it is not clear how they calculated the correlations (Pearson or Spearman, or even Kendall’s Tau) and on what exactly. This analysis would be with only 10 samples or just 5 when sex is differentiated. This is too small sample size to draw conclusions.

In particular, the donors participating in the study have not been sufficiently characterized immunologically (Ayuk et al. 2025), for example compared to Dörnte et al. (2022)—a study by the manufacturer of the peptide pool used. The study by Ayuk, et al. reveals significant differences among vaccine blood donors in terms of repeated interaction between the spike protein and immune cells (exposure), and the subsequent stimulation of these cells by the SARS-CoV-2 spike protein following vaccination and/or infection. In the two small comparison groups of blood donors (female and male), the number of different SARS-CoV-2 spike protein exposures—i.e. immunizations from vaccines and SARS-CoV-2 infections—ranges from two to seven (Ayuk et al. 2025). This raises the key immunological question of whether, for example, a woman who has been exposed to the spike protein twice (two-dose vaccination) would, in principle, exhibit a different T cell and B cell response to a man who has been exposed to the protein (or its peptides) seven times (four-dose vaccination and three infections). This should by no means be overlooked in the context of the statements and speculations made regarding SARS-CoV-2 vaccinations and potential PFAS effects, taking sex-specific differences into account. Similarly, different vaccination patterns and disease progression in SARS-CoV-2 must be taken into account, irrespective of sex-specific differences. Furthermore, unlike to Dörnte et al. (2022), there exists a lack of relevant information regarding exactly when each individual had their last immunologically significant exposure to the SARS-CoV-2 antigenic protein prior to the start of the study.

Both factors—varying numbers of exposures and unclear time frames since the last exposure—could undoubtedly lead to different T cell immune responses induced by the SARS-CoV-2 peptide pool; these are not comparable and also raise questions regarding the sex-specific differences postulated here.

Given the small number of vaccinated blood donors and the insufficiently documented history of previous vaccinations (and infections), we do not consider it is possible to draw any immunologically reliable conclusions regarding sex-specific NK-, T- and B-cell-mediated immune responses to the SARS-CoV-2 vaccine, or regarding the possible effects of PFAS on vaccine efficacy. It is well established that factors such as timing of administration, antigen concentrations (see below), adjuvants, pharmacological formulations and the recipient’s HLA system influence vaccine responses (Dornte et al. 2022, 2025; Knierman et al. 2020; Strazar et al. 2023). To draw evidence-based conclusions, it is therefore not sufficient merely to state that all participants were vaccinated “at least twice” with the COVID-19 mRNA vaccines and had “similar time intervals” between doses. More detailed information is lacking. Conclusions regarding NK or T cell (memory) responses, or regarding potential antibody responses (titers), which were not investigated in this study, must therefore be regarded as speculative.

#### Kinetics and conditions of SARS-CoV-2 peptide-pool induced human T cell activation

Many molecular responses of human T cell (sub)populations triggered by antigen-specific peptides are governed by well-established kinetic principles and the concentrations of specific antigenic stimuli; this also applies to SARS-CoV-2. The activation protocol described here (Ayuk et al. 2025) differs from numerous, very similar T cell activation protocols for SARS-CoV-2-specific T cell responses that have already been described in detail (Table 1). Neither are these standard publications cited, nor are reasons given as to why this alternative approach—with regard to the specific kinetic profile of the T cell activation markers—was adopted here. In total there was an overall incubation time of greater than 60 h, consisting of a 24-h in vitro stimulation of human PBMC with the peptide pool, after overnight incubation of PBMC (> 12 h), and after an additional 24-h chemical PFAS stimulation that included IL-2 (300 U/mL) and anti-CD28 (6 µg/mL). In comparison, Neidleman et al. (2021) used overlapping peptide pools of the spike protein to measure intracellular IFN-γ concentrations calculated as antigen-specific T cell responses after 6 h of stimulation with overlapping 15-mer peptides. In another study (Oberhardt et al. 2021), following in vitro expansion intracellular IFN-γ was measured after restimulation for 5 h (plus Brefeldin A). Similarly, Dörnte et al. (2022) measured intracellular IFN-γ, TNF-α, and IL-2 after a 6-h stimulation as mentioned, with a 4-h Brefeldin A incubation included.

**Table 1:** Methods for the detection of antigen-specific CD154+ T cells following stimulation with virus-specific peptide pools

Study peptide pool (supplier)	Use of frozen cells	Preincubation*	Serum	IL-2**	Individual peptide concentration	Antigen stimulation time	Well plate format
Frentsch 2005 CMV pp65 (Jerini)	−	−	Human AB	−	4 μg/mL	6 h	Flat bottom
Braun 2020 SARS-CoV-2_S (JPT)	−	−	Human AB	−	1 μg/mL	16 h	Flat bottom
Saggau 2022 SARS-CoV-2_S (JPT)	−	−	Human AB	−	0.5 μg/mL	6 h	Flat bottom
Dörnte 2022 SARS-CoV-2_S (Miltenyi Biotec)	−	−	Human AB	−	1 μg/mL	6 h	Flat bottom
Ayuk 2025 SARS-CoV-2_S (Miltenyi Biotec)	+	24 h***	**Fetal bovine**	**300 U/mL (30 ng/mL)**	2 μg/mL	24 h***	U-bottom
Peptivator data sheet SARS-CoV-2_S (Miltenyi Biotec)	(+)	−	Autologous or human AB	−	1 μg/mL	6 h****	Flat bottom

Key methodological parameters from various studies are listed. “−”—Not used/not done, “+”—Used/done, S—Spike protein of the SARS-CoV-2 virus, CMV pp65—Phosphoprotein 65 of the human cytomegalovirus, (+)—“best results with fresh cells”. Particularly significant deviations between the Ayuk 2025 study and the other studies are highlighted in bold

*Additional pre-incubation besides an optional overnight incubation before stimulation in the next morning

**During antigen stimulation of T cells

***24 h PFAS incubation followed by 24 h SARS-CoV-2 peptide pool stimulation (total time before FACS measurement 48 h)

****Intracellular cytokine staining after Brefeldin A addition for the last two hours; CD154 MicroBead kit indicates incubation from 4 to 16 h

The importance of the kinetics in monitoring T cell activation has been demonstrated for several parameters, including the upregulation of CD154 (CD40-L). CD154 represents an important activation-associated marker following human antigen-specific T cell stimulation, usually upregulated rapidly, after 5 to 7 h of PBMC stimulation, as exemplified in responses to *Staphylococcus aureus* enterotoxin B (SEB) and the SARS-CoV-2 spike protein or metal allergens (Aparicio-Soto et al. 2020; Dornte et al. 2022; Frentsch et al. 2005; Saggau et al. 2022). Thus, antigen-specific CD154 expression is typically detectable between 4 and 12 h after (re-)stimulation of CD4+ T cells (Bacher and Scheffold 2013), and becomes increasingly unreliable after 24 h of stimulation (or 48 h, if PFAS stimulation is included), as confirmed in this study by unchanged CD154 expression (Ayuk et al. 2025).

However, the absence of changed CD154 expression after 24 h does not rule out the option that T cell-associated cytokine expression—as measured in this study—could also occur independently of specific activation of SARS-CoV-2-specific T cell receptors (TCR) by the peptide pool. For example, this could occur via IL-12 from chemically co-stimulated APCs/monocytes, which could lead to altered IFN-γ responses (Avice et al. 1998). Thus, the presented data do not show an unambiguous direct correlation between CD154 activation marker expression and IFN-γ production, as has already been demonstrated in many other studies on SARS-CoV-2-associated activated T cells (Dornte et al. 2022; Saggau et al. 2022). Yet, because CD154 is only transiently expressed after T helper cell activation, possible SARS-CoV-2-specific T cell activation is not excluded in the study presented. As mentioned earlier, unlike in Saggau et al. (2022), no control peptide pool from a virus other than SARS-CoV-2, such as influenza A, was included. Interestingly, non-TCR-induced expression of CD154 and CD69 stimulation markers had already been demonstrated previously (Frentsch et al. 2005). Furthermore, the addition of relatively high concentrations of IL-2 (300 U/mL) by Ayuk et al. (2025) could likely mask the SARS-CoV-2-specific TCR signals and cytokine release, and could also affect B cells, NK cells, and monocytes. Addition of IL-2 addition during peptide stimulation contradicts the approaches used by LeBert et al. (2020), in which SARS-CoV-2T cell lines were expanded in the presence of 20 U/ml recombinant IL-2, as well as those of Saggau et al. (2022), in which, following the expansion of antigen-specific T cell lines (with 200 U/ml IL-2, Proleukin, Novartis) and the restimulation of antigen-reactive T cells, IL-2 was explicitly removed for 2 days prior to SARS-CoV-2 peptide pool-specific restimulation (Table 1). Since the activity of the recombinant human IL-2 protein (from PeproTech®) used in this study is 300 U/ml (compared to supplier’s data sheet), this corresponds to a concentration 50% higher than the dose used for the expansion of SARS-CoV-2-specific T cell lines, and contrasts with the specific T cell restimulation reported by Saggau et al. (2022) as well as the short-term stimulation reported by Dörnte et al. (2022).

#### Re-stimulation of SARS-CoV-2-reactive T cells

To re-stimulate SARS-CoV-2-reactive T cells, Dörnte et al. (2022) as well as Saggau et al. (2022) incubated freshly isolated human PBMCs with 5% human AB serum, while Ayuk et al. used previously cryopreserved (in 90% foetal bovine serum, plus 10% DMSO) PBMCs in 10% foetal bovine serum (FBS). However, compared to the use of human serum, incubation with FBS may lead to non-specific stimulation and affect virus-specific T cell reactivity and IFN-γ secretion (Adhikary et al. 2008). According to the manufacturer’s instructions, the ‘best results’ in cytokine secretion by SARS-CoV-2-specific T cells are achieved by stimulating freshly isolated PBMCs, although frozen PBMCs are also possible. In addition, the culture medium for restimulation of antigen-specific T cells should contain ‘5% human serum, e.g. autologous or AB serum’ (PepTivator® SARS-CoV-2 Prot_S Complete, Miltenyi Biotec).

In addition, the concentration of peptides used by Ayuk et al. is higher than in other studies. Dörnte et al. (2022) re-stimulated the T cells with a final concentration of approximately 1 µg/ml of each SARS-CoV-2-derived peptide in the peptide pool for 6 h, Braun et al. (2020) used peptide pools with 1 µg/ml per peptide, and Saggau et al. (2022) used 0.5 µg/ml per peptide and lower concentrations to evaluate functional avidity (Table 1). Ayuk et al. used a concentration of 2 µg/ml of SARS-CoV-2 peptides, at least twice as high in comparison to the many studies already conducted with the SARS-CoV-2-specific peptide pools (Dornte et al. 2022) or as recommended by the manufacturer (Miltenyi 2021).

### Methodology and data analyses

The main methodological limitations of the study relate to the processing of the fluorescent activated cell sorting (FACS) data, and the statistical methods used.

#### Omission of FACS data

In this study, authors did not disclose original FACS data of absolute cell counts of PFAS- and SARS-CoV-2-peptide-pool treated cells—after 24-h (PFAS) and 48-h stimulation (PFAS plus peptide pool), including data of all cytokine concentrations generated by FACS-based immunophenotyping and LEGENDplex™ bead-based immunoassay analyses. Compared with the absolute cell counts for SARS-CoV-2 reactive CD4+ T cells reported by Saggau et al. (2022), it is unclear whether the authors calculated the FACS data based on 25 or 5,000 absolute cell counts. Additional important controls are missing, such as control activation of PBMC without DMSO, and testing of reference molecules, such as stimulation by lipopolysaccharide (LPS) or by another non-related peptide-pool as negative control like influenza A or CMV (PepTivator Influenza A or PepTivator CMV, Miltenyi Biotec), as shown by Saggau et al. (2022). The supplementary figures also lack examples of intracellular staining as well as other data such as the percentage of IFN-γ-positive cells compared to an isotype antibody control. Furthermore, since all values are reported as fold-change relative to the DMSO controls, it is unclear what the actual percent positive values are for cell-based population analysis or, in the case of cytokine levels, what the absolute concentrations are in pg/mL. It is impossible to know if, for example, a 1.2-fold change in a cell population is from 0.1 to 0.12% or from 5 to 6%. Similar concerns exist for the fold-changes in cytokine levels shown.

Immunophenotyping has shown that rising serum levels of perfluorooctanoic acid (PFOA) and perfluorooctane sulfonic acid (PFOS), among others, may correlate with a decrease in monocytes and natural killer cells (NK cells), and possible necrotic/lytic cell death or apoptosis in human or murine immune cells (McCall et al. 2024; Zhang et al. 2013). It is therefore difficult to integrate the results from this study into described PFAS effects that correlate with reduced IFN-γ production (immunosuppression) or cell death (Abraham et al. 2020; Phelps et al. 2024). We do concur with Tan et al. (2023), who investigated the association between PFAS and serum cytokine concentrations in a mother–child cohort and demonstrated a strong association between PFAS mixtures and IFN-γ. However, given the heterogeneity of study designs it remains challenging to assess whether or not there is a consistent pattern for the varying expression of inflammatory markers such as IFN-γ following PFAS exposure, and to draw conclusions on a causal relationship between PFAS exposure and human immune health effects (Antoniou and Dekant 2024; Tan et al. 2023).

#### Statistics

Throughout the paper, Ayuk et al. represented the data (concentrations of PFAS-treated samples) as fold-changes in relation to the DMSO controls measured from each donor in each analysis, which they have set to be 1 in all samples (Ayuk et al. 2025).

This approach poses significant methodological issues, when it comes to evaluating the statistical significance of the effect of the tested PFAS concentrations (1×, 10×, 100×, 1000×).

Indeed, the inter-individual variability of the DMSO controls is deliberately obscured, resulting in a substantial loss of information, which is problematic for statistical significance, and in favor of finding significant differences systematically. If the statistical comparisons used are invalid, the interpretations and conclusions are unsupported.

Furthermore, statistical comparisons in the supplemental data (comparing Dunnett’s and Bonferroni’s among others) need an independent control also accounting for variations as they are meant to compare two or more distributions. In our opinion, both are wrongly applied since they are using the DMSO fold-change values with zero variation as a control comparison.

#### PFAS mixtures used in this study

The PFAS mixture used (1×) in this study represents plausible values for the main compounds of internal PFAS exposure. However, it should be noted that the blood levels used are based on Scandinavian studies from 1996 to 2004 (Supplementary materials, Table S8 Levels of perfluorinated compounds in blood from Scandinavian studies (Berntsen et al. 2017)). The authors did not take into account that serum concentrations of some PFAS, particularly PFOS, are declining in the population (Sonnenberg et al. 2023) and that more recent data on internal exposure indicate low serum concentrations, particularly of PFOS, but also of PFOA (Duffek et al. 2020). It should also be emphasized that in the study, the effects described in Sect. 3.1 (PFAS mixtures effect on innate immune response following SARSCoV-2 spike peptide stimulation, in Ayuk et al. 2025) were not observed at concentrations relevant to humans at real life concentrations (1×), as it is suggested in the study’s abstract, but may occur at higher, non-real life PFAS concentrations. Consequently, the PFAS concentrations used in this study do not correspond to the current level of exposure in the population, and the effects observed only occurred at significantly much higher, non-physiological concentrations. Although the limitation is addressed in the discussion section of the study, it is not reflected in the key statements on the PFAS mixture in the abstract of the article. The study thus attempts to demonstrate ‘significant immune response values’ for PFAS concentrations that do not occur in reality (1,000 times higher), as perhaps one or two out of five donors are highly reactive (concerning CD4+ and CD8+ T cells, and IFN-γ induction by 100× and 1000× PFAS concentrations (Ayuk et al. 2025)). Furthermore, the DMSO control (see above) is questionable, and DMSO could also influence individual, unanalyzed, pre-existing PFAS concentrations in the blood; in addition, the absolute numbers of (non-) reactive immune cells are unknown (not specified in the publication).

Since both publications (Ayuk et al. 2025; Berntsen et al. 2017) report identical PFOS concentrations (stock solution), Ayuk et al. most likely used the same mixture that Berntsen et al. had prepared in 2017, particularly, as from a chemically perspective, it would be very difficult for a laboratory to produce exactly the same validated physical PFAS mixture twice. This raises the question of whether the individual chemicals in the mixtures still retain their chemical and immunobiochemical properties after more than seven years of storage; no mention was made of any corresponding quality control measurements. Since PFOS accounts for around 75% of the mixture and PFOS can bind to serum albumin, the question arises as to whether some of the observed effects might also be influenced by variations in the serum concentrations used (Yang et al. 2023). A control group only treated with PFOS would have been very useful for the study, as would information on the PFAS concentrations in the blood of individual donors.

#### Specific T cell activation by the PepTivator® SARS-CoV-2 Prot_S Complete peptide pool in the study

In this approach, Ayuk et al. (2025) examined epitope-specific immune responses from donors who had previously been vaccinated with SARS-CoV-2. The key event in this model is the stimulation of human T cells by the PepTivator® SARS-CoV-2 Prot_S Complete peptide pool (6 nmol/peptide; lyophilized peptides with stabilizer; average purity of peptides > 70%; #130-127-951, Miltenyi Biotec, Bergisch Gladbach, Germany), which was “specifically developed for efficient in vitro stimulation of antigen-specific CD4+ and CD8+ T cells” (Miltenyi 2021), as the solution consists (mainly) of overlapping peptides with 15-mer sequences (with 11 amino acids overlap). This, according to the manufacturer, covers the entire protein-coding sequence (amino acid (aa) 5-1273; excluding aa 1–4 signal peptide) of the spike glycoprotein (‘S’) of SARS-CoV-2. Thus, it represents a standard format for peptide pools to activate antigen-specific T cells (Chevalier et al. 2015). This spike molecule is responsible for the recognition and binding of the virus to the host cell and is used as the target protein for vaccination (Bai et al. 2022; Feikin et al. 2022; Hoffmann et al. 2020). In this study, the authors did not take into account a groundbreaking publication by the manufacturer on the stimulation of T-memory cells in the SARS-CoV-2 vaccination (Dornte et al. 2022), nor did they consider the data sheet for the peptide pool. Otherwise, they would have realized that the peptide pool they used consisted of 360 different peptides; and they would not have consistently used the term ‘peptide’ instead of ‘peptides’ or ‘peptide pool’. This leads to uncertainty as to whether a single peptide or the entire peptide pool was used, and thus to potential misinterpretations of the immunological aspects of the entire experimental design and the conclusions drawn from it.

## Conclusions

The potential immunomodulatory effects of PFAS on SARS-CoV-2-specific memory T cells need to be investigated in detail. The study by Ayuk et al. addresses a crucial question regarding the immunological protection offered by a SARS-CoV-2 vaccine in relation to toxicologically active chemicals such as PFAS, which are widespread in our environment and can also be detected in human blood.

In our view, the study has significant limitations: Firstly, with regard to the methodology used in the application of the antigen/SARS-CoV-2-specific peptide pool for the activation of polyclonal T cells, which deviates from the manufacturer’s instructions and other key reference studies. Not only are FACS control measurements and the presentation of raw data (e.g. as a supplement) missing from this key method, but the statistical approach also raises fundamental questions.

Secondly, the design of the vaccination study investigating adaptive T cell memory responses to SARS-CoV-2 under the influence of PFAS following at least two vaccinations in blood donors must be fundamentally questioned. Compared to reference studies, the recruited blood donors are inadequately characterized and represent a highly heterogeneous group immunologically, with between two and seven possible molecular exposures to the spike protein antigen (or peptides thereof), either through vaccination or infection. Furthermore, the time intervals between these molecular SARS-CoV-2 protein or peptide (pool) exposures varied greatly, including in relation to the start of the study. In addition, the study used blood cells that had previously been frozen and exposed to bovine serum rather than human serum, as well as high concentrations of IL-2.

Although established, specific SARS-CoV-2 protocols for assessing an activated T cell memory response in human blood were available, neither their kinetics nor the relevant T cell receptor-associated activation markers were taken into account, nor were these studies discussed in the present study.

A critical analysis of the data—such as the small number of blood donors and their variable or unknown immune status with regard to SARS-CoV-2 immunity—does not, in our view, permit any evidence-based conclusions at this stage regarding an immunomodulatory NK-, T- or B-cell effect of a PFAS mixture, which only demonstrated effects at concentrations that are not expected to be found in the human blood based on currently known exposure levels.

## Data Availability

No datasets were generated or analysed during the current study.

## Abbreviations

BfR: German Federal Institute for Risk Assessment
CD154: Ligand for CD40 (CD40L)
EFSA: European Food Safety Authority
FDA: The U.S. Food and Drug Administration
IFN-γ: Interferon gamma
PBMC: Peripheral blood mononuclear cells
PFAS: Per- and polyfluoroalkyl substances
SARS-CoV-2: Severe acute respiratory syndrome corona virus 2
